# Process Model Approach to Predict Tablet Weight Variability for Direct Compression Formulations at Pilot and Production Scale

**DOI:** 10.3390/pharmaceutics13071033

**Published:** 2021-07-07

**Authors:** Raghu V. G. Peddapatla, Gerard Sheridan, Conor Slevin, Shrikant Swaminathan, Ivan Browning, Clare O’Reilly, Zelalem A. Worku, David Egan, Stephen Sheehan, Abina M. Crean

**Affiliations:** 1 SSPC Pharmaceutical Research Centre, School of Pharmacy, University College Cork, T12 K8AF Cork, Ireland; r.peddapatla@umail.ucc.ie (R.V.G.P.); a.crean@ucc.ie (A.M.C.); 2 Alkermes Pharma Ireland Limited, N37 EA09 Athlone, Ireland; gerrysheridan1990@gmail.com (G.S.); conorslevin93@hotmail.com (C.S.); ivanbrowning1@gmail.com (I.B.); Clare.OReilly@helsinn.com (C.O.); Zelalem.Worku@alkermes.com (Z.A.W.); 3 Pharmaceutical Manufacturing Technology Centre (PMTC), Bernal Institute, University of Limerick, V94 T9PX Limerick, Ireland; david.egan@ul.ie; 4 Alkermes Inc., Waltham, MA 02451, USA; Shrikant_Swaminathan@amat.com

**Keywords:** quality by design, tablet weight variability, powder flow, process optimization, process model, direct compression

## Abstract

Optimizing processing conditions to achieve a critical quality attribute (CQA) is an integral part of pharmaceutical quality by design (QbD). It identifies combinations of material and processing parameters ensuring that processing conditions achieve a targeted CQA. Optimum processing conditions are formulation and equipment-dependent. Therefore, it is challenging to translate a process design between formulations, pilot-scale and production-scale equipment. In this study, an empirical model was developed to determine optimum processing conditions for direct compression formulations with varying flow properties, across pilot- and production-scale tablet presses. The CQA of interest was tablet weight variability, expressed as percentage relative standard deviation. An experimental design was executed for three model placebo blends with varying flow properties. These blends were compacted on one pilot-scale and two production-scale presses. The process model developed enabled the optimization of processing parameters for each formulation, on each press, with respect to a target tablet weight variability of <1%RSD. The model developed was successfully validated using data for additional placebo and active formulations. Validation formulations were benchmarked to formulations used for model development, employing permeability index values to indicate blend flow.

## 1. Introduction

Direct compression is a relatively simple method to manufacture pharmaceutical tablets. It involves two primary processing steps, viz., blending and compaction and is less complicated compared to tablet production involving a dry granulation or wet granulation step [1]. The compaction process can be subdivided into die filling, compaction, decompression and ejection. To produce tablets of sufficient quality at production scale, direct compression formulations are required to exhibit adequate flow, blend uniformity, compaction and ease of ejection from the tablet press [1,2,3,4,5].

Formulation flow properties significantly impact on the first stage of compaction, i.e., die filling. Die filling determines the mass of formulation in the die and hence tablet weight. For uniform blends, the drug dose per tablet is determined during the die filling step. Non-uniform die filling can result in variable tablet density/porosity, hardness/tensile strength, disintegration and dissolution [6,7,8,9,10,11]. To facilitate uniform die filling, rotary tablet press designs incorporate a forced feeder, or feed frame. The design of the feed frame varies between tablet press models [9]. Commercial tablet processes aim to maximise the tablet production rate while maintaining compliance with product specifications. The relationship between press speed, feed frame design and speed, and formulation flow properties influences the die filling process and hence the quality of the tablets produced [9,12].

The regulatory guidance regarding the application of Quality by Design (QbD) during the design and development of pharmaceutical processes, ICH Q8 (R2), was introduced more than 10 years ago [13]. Process understanding and optimisation are integral to QbD. The establishment of optimum processing conditions for direct compression tablet formulations is widely reported [14,15,16,17,18,19,20,21]. The majority of these studies were completed using laboratory- or pilot-scale tablet presses with a limited number at commercial scale [20]. It is worth noting that for multi-stage processes such as direct compression, the process can be broken down into a series of process optimizations for various stages: filling, compaction and ejection. For example, optimum process conditions developed for the die filling stage of a direct compression process would identify tablet weight and tablet weight variability as critical quality attributes and investigate material attributes, such as blend density and flow, and processing parameters, such as press speed and feed frame speed, to determine optimum processing parameters [6,9,22,23]. For any formulation, a tablet’s weight uniformity depends on the uniformity of the die cavity fill. The efficiency of the die filling process is influenced by powder blend properties such as flowability, cohesion, particle size, morphology, as well as process parameters such as die table and feed frame speed [24].

The optimum process parameters are commonly established during the development stage using pilot-scale tablet presses due to limited access to production equipment and the quantities of drug required at early stages of development [25,26]. However, due to differences in equipment models/designs utilised at pilot scale and at production scale, extrapolation of process understanding from pilot to production scale is challenging, as this may be scale- and equipment-dependent. To employ the process conditions at commercial scale that are developed at pilot scale, a justification is required: geometric considerations, kinematic considerations, heat and mass transfer, or dimensionless numbers [27]. It is challenging to generate a single optimum processing condition that will encompass both pilot- and production-scale equipment. This is particularly evident for tablet presses where feeder designs and press speeds can vary greatly between tablet presses from different manufacturers [28].

Additionally, achieving optimal processing parameters can be formulation-dependent, and the transfer of learnings between formulations is challenging. The ability to predict the performance of formulations and establish optimum processing conditions on different production press models is advantageous. It reduces costly experimentation during scale-up, reduces the risk of failure on scale-up and transfer between equipment trains and enables flexibility in production schedules. However, due to differences in press designs and control strategies, it is challenging to transfer a formulation between production presses. Each process optimization generated is to some degree limited by the equipment on which the experimental runs were conducted and the formulation processed.

The study presented details the development of an empirical process model for the die fill filling step which describes the tablet weight variability of three placebo formulations, with different flow characteristics, on three different tablet presses. The tablet presses used to establish the process model included one pilot-scale and two production-scale presses. The model developed in this work established the optimum processing parameters of each formulation on each press with respect to tablet weight variability. The model was successfully validated by retrospectively predicting the behaviour of an additional placebo and API formulation on the production-scale tablet presses.

The aim of the study is not to compare the capability of different tablet press designs at comparable process settings. The study was designed to evaluate the tablet weight variability for blends with a range of flow properties on tablet presses equipped with their standard feeder systems and employing control strategies routinely using in commercial production. The benefit of such an applied approach is that it can inform the manufacturer on how formulations will behave in practice during transfer between presses and scale-up from pilot level to production level.

## 2. Materials and Methods

### 2.1. Formulation Design

The three placebo formulations used in the development of the process model were designed to exhibit good, fair and passable flow. Carr’s compressibility index [29], Hausner ratio [29,30], angle of repose and permeability index (detailed in Section 2.3) were determined for the three formulations as markers of powder flowability and used to classify blend flow. The composition of each formulation and its flow characteristics are described in Table 1. With the exception of sucrose octaacetate, all excipients employed are commercial excipient grades meeting the excipient monograph listed in the USP-NF. Sucrose octaacetate was included to mimic the presence of a drug substance which would inhibit blend flowability. It was included in the fair flow blend at a concentration of 20% *w*/*w* and in the passable flow blend at a concentration of 40% *w*/*w*. Formulation 1 was designed to exhibit good flow, formulation 2 to achieve fair flow, and formulation 3 to achieve passable flow.

### 2.2. Formulation Preparation

Total weight of each blend prepared was approx. 75 kg. Excipients were dispensed according to the formulation type (Table 1) and passed through a 450 µm sieve (Sweco Europe S.A., Nivelles, Belgium) to remove agglomerates. With the exception of magnesium stearate, all excipients were added into a 100 L IBC (Intermediate Bulk Container, Coleshill, UK) which was attached to the drive of the Blender unit (Pharmatech, Coleshill, UK) via a clamping system. The IBC was rotated for 18 min at 20 rpm, for a total of 360 revolutions. Magnesium stearate was then added to the other components in the IBC and blended for an additional 3 min at 20 rpm, for a total of 60 revolutions.

### 2.3. Tablet Blend Permeability

Permeability of each formulation blend was measured using a Freeman FT4 Powder Rheometer (Freeman Technology Ltd., Tewkesbury, UK). Blend samples were poured into a 25 mm-diameter (10 mL) splitting cylindrical vessel. A 23.5 mm helical blade was inserted and rotated in the powder bed to condition the powder, removing excess air. Following conditioning, the vessel was split, and the mass recorded. Permeability, *k*, was measured as pressure drop across powder beds against a varying applied normal pressure (1–15 kPa), while the air velocity through the powder bed was maintained at constant rate of 2 mm/s. The greater the pressure drop, the less permeable the powder. Permeability was derived using Darcy’s law, from Equation (1) [31]:

(1) $$k=\frac{q.µ.L}{\Delta P}$$

where *k* is the permeability (cm^2^), *q* is the flow rate of air (cm/s), *µ* is the viscosity of air (Pa·s), *L* is the length of the powder bed (cm), and ΔP represents the pressure drop across the powder bed (mbar).

### 2.4. Tablet Production

Tablets of each formulation blend were produced on one pilot-scale rotary tablet press (KG RoTab (KG-Pharma, Scharbeutz, Germany)) and two production-scale rotary tablet presses (Fette 1200i (Fette Compacting, Schwarzenbek, Germany) and Modul^TM^ P (GEA, Bergensesteenweg, Belgium)). Some key technical differences between the tablet presses selected, including tooling details, relevant to this study are listed in Table S1. KG RoTab and Fette 1200i were operated using main compression force control, i.e., a specified range was set for the main compression force and if it deviated outside this specified range, the tablet filling depth was adjusted accordingly. Module P tablet press was operated in mode 2, which involves weight control, by measuring punch displacement during precompression.

Tablet blends were compressed using shield-shape punches to a target weight of 240 mg. A constant pre-compression force of 1 kN was used on all tablet presses. The main compression force was optimised from the compression profile of each formulation on each tablet press to achieve a nominal target porosity of approx. 15%. To achieve the target porosity for different formulations, different compression forces were used. This data is included in supplemental data, Table S2. Feeder and press speed were key press process parameters investigated in this study and varied across runs based on the design of the experiments described in Section 2.5.

### 2.5. Design of Experiments

A 12-run full response surface design with 4 centre points (2^3^ factorial DoE) was used to study each formulation, on each tablet press. Overall, 12 × 3 × 3 runs were completed (108 runs). The design of experiments (DoE) was carried out using JMP statistical software (Version 13, SAS Institute, Inc., Cary, NC, USA). The cube plot and DoE run order of experiments for three different formulations on three tablet presses is shown in supplementary information (Figure S1 and Table S3). The objective of the DoEs was to identify optimum feeder speed and press speed combinations for each blend on each tablet press design to inform the achievable production rates which produced a tablet weight variability <1% RSD. The feeder speed rates investigated were equivalent for each press, as shown in Table 2. However, it is important to note that due to differences in feeder designs on each press, the feeding processes were not comparable.

Tablet press speed levels varied across the tablet press models based on their respective minimum and maximum press speeds. For each tablet press, at least one press speed level was set to match another press based on their dwell times, as shown in Table 2.

### 2.6. Measurement of Tablet Weight Variability and Porosity

Approximately 3 kg of blend was compacted for each run of the DoE. Twenty tablets were collected after every 1 kg of blend compacted, and weights were measured using a Smart-test 50 Autotester (Pharmatron, Aesch, Switzerland). Mean tablet weight and tablet weight variability (%RSD) were measured for every 20 tablets collected per 1kg blend compacted. Tablet porosity was measured using equation 2:

(2) (1−Tablet Solid Fraction)×100

### 2.7. Data Analysis

Process data (tablet press, feeder speed, press speed), mean tablet weights and tablet weight variability from the independent DoEs executed for each formulation/press combinations were imported and analysed using JMP13 statistical software (SAS, Cary, NC, USA). This data was integrated in JMP software to develop a model which combined the continuous variables (press speed and feeder speed) with categorical variables (formulation and press type). Tablet press speed was standardized for each tablet press by subtracting the mean press speed from the actual press speed and dividing by the standard deviation. The standardised press speeds were referred to as the ‘Coded’ press speeds. All continuous factors were centred during the analysis. A full factorial model considering linear, 2-way, 3-way, 4-way interactions and quadratic terms was created using fit model analysis in JMP. The model identified statistically significant factors (process parameters, formulation type and tablet press) effecting tablet weight variability (%RSD) for the three formulations on the three tablet presses.

## 3. Results

### 3.1. Blend Characterisation

Carr’s index, Hausner ratio, angle of repose and permeability analysis were performed on tablet blend formulations to confirm differences in flow behaviour prior to compaction and to classify blends. Formulation 1 exhibited the best flow characteristics, with the lowest Carr’s index, Hausner ratio and angle of repose and the highest permeability index. Formulation 3 exhibited the poorest flow characteristics, with the highest Carr’s index, Hausner ratio and angle of repose and the lowest permeability index. Formulation 2 showed flow characteristics between those of formulations 1 and 3 (Table 1). The various powder flow indices distinguished differences between the three formulations. Based on Carr’s index and Hausner ratio values, formulation 1 was categorised as good flow. Formulations 2 and 3 were categorised as fair- flow and passable- flow, respectively, based on Carr’s index, Hausner ratio and angle of repose values (USP, <1174> Powder Flow).

### 3.2. Tablet Weight Variability (%RSD)

**Figure 1:**
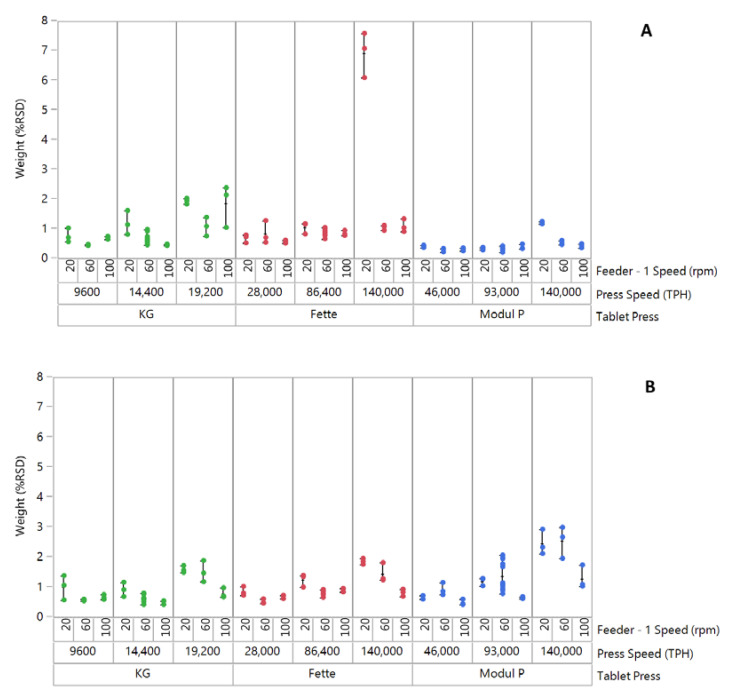

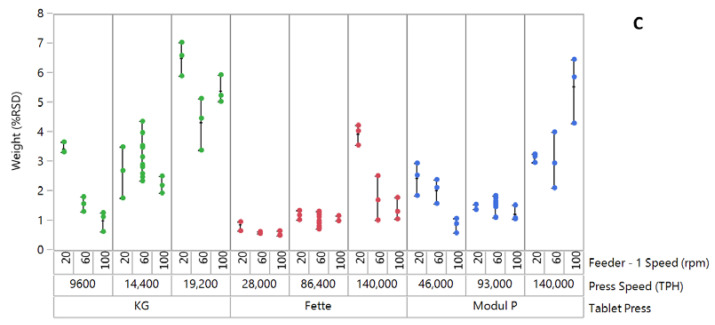
Plot of tablet weight variability (%RSD) for three formulations compacted on three tablet presses. (**A**) Good-flow formulation, (**B**) Medium/fair-flow formulation and (**C**) Passable-flow formulation. Each point on the graph signifies % RSD for *n* = 20 tablets. Approximately 12,000 tablets were compacted for individual runs in a DoE. A total of three in-process checks (IPCs) were performed for 20 tablets after every 4000 tablets compacted, within an individual DoE run. Data point for good flow formulation on Fette 1200i at 20 rpm feeder speed/140,000 TPH press speed was excluded in process model development.

The influence of feeder speed and press speed on tablet weight variability (%RSD) for formulation 1 “good flow” across three tablet presses is shown in Figure 1A. Good control of tablet weight variability can be observed across all three tablet presses, with the Modul P showing the lowest tablet weight %RSD across different feeder speeds and press speeds (Figure 1A). The exception to the trend can be seen for the combination of lower feeder speed and higher press speed on the Fette 1200i. At these process settings, a large increase in tablet weight variability was measured. It was also noted that for this setting, there was a reduction in average tablet weights (221.1 ± 2.84 mg) and failure to achieve the target tablet weight of 240 ± 18 mg. At all other settings, the target weight specification was achieved for this formulation. The high tablet weight variability was attributed to starving of dies at the lower feeder speed and high press speed, which was not sufficient to feed enough material into the dies reproducibly.

The effect of feeder speed and press speed on the tablet weight variability (%RSD) of formulation 2 ‘fair flow’ is shown in Figure 1B. Across all three tablet presses, an increase in tablet weight variability was observed at the lower feeder speed setting, with increase in tablet press speed from lowest to highest. This trend was more pronounced on Modul P tablet press. Across all presses, the average %RSD exceeded the nominal acceptable commercial limits of 1% for runs at the highest press speeds. Formulation 3 “passable flow” exhibited the highest tablet weight variability (%RSD) of the three blends on all three presses (Figure 1C). Greatest variability was measured on the pilot-scale KG RoTab press. Variability was greatest at the highest press speed on all three presses.

### 3.3. Process Model to Predict Tablet Weight Variability (%RSD)

A full factorial model was investigated to identify the significant effects of all factors and their interactions (linear, 2-way, 3-way, 4-way interactions and quadratic) on tablet weight variability (%RSD). The fit model analysis predicted the best fit for the log-transformation of weight variability. The full factorial model obtained was statistically significant (*p* < 0.0001), with ***R***^2^ of 0.8619 and low root-mean-square error. A reduced model was fit by reducing terms to factors with a p-value less than 0.05; non-significant factors were removed. A summary of the statistical parameters for the reduced regression model is shown in Table 3. Effect analysis for log-transformed tablet weight variability (%RSD) is shown in Table 4. Tablet press, formulation type, feeder speed and press speed showed critical effects on tablet weight variability.

It is not surprising that the four factors studied were significant individually, as they were selected because they are widely reported and known for their impact on weight variability. The summary of individual factors and their effects on weight variability is shown in supplementary information, Figure S2. An interactive prediction profiler was generated based on the model developed, to understand the effect of process factors on weight variability. An example of the prediction profiler is shown in supplementary information, Figure S3.

### 3.4. Process Optimization for Tablet Weight Variability (%RSD)

**Figure 2:**
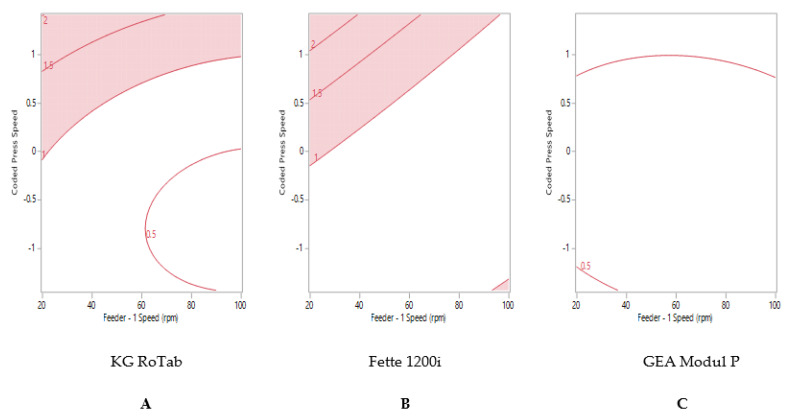
Two-dimensional contour profile showing optimum tablet press speed and feeder speed settings (uncoloured) for formulation 1 “good flow” to achieve tablet weight variability ≤1% RSD on (**A**) KG RoTab, (**B**) Fette 1200i and (**C**) Modul P. Coded press speeds refer to standardized press speed for each tablet press by subtracting the mean press speed from the actual press speed and dividing by the standard deviation. Refer to supplementary information (Table S4) for the actual press speeds.

The reduced model obtained was used to establish optimum process parameters for acceptable weight variability (%RSD). The optimum process parameters were established to achieve a target % RSD between 0% and 1% for each formulation on each tablet press. Figure 2 shows the contour or sweet-spot profile for formulation 1 ‘good flow’, where the regions uncoloured indicate the optimum process conditions to achieve ≤1% RSD. At higher tablet press speeds, higher feeder speeds were required to attain reduced tablet weight variability for both production presses (Fette and Modul P), Figure 2B,C. The maximum press speed at which ≤1% RSD tablet weight variability could be attained on the pilot-scale press (KG RoTab) was 17,800 TPH (coded press speed 1) (Figure 2A).

**Figure 3:**
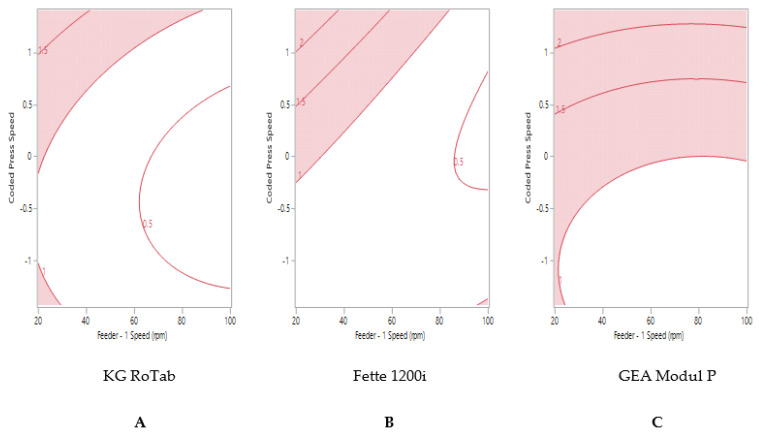
Two-dimensional contour profile showing optimum tablet press speed and feeder speed settings (uncoloured) for formulation 2 “fair” to achieve tablet weight variability ≤1% RSD on (**A**) KG RoTab, (**B**) Fette 1200i and (**C**) Modul P. Coded press speeds refer to standardized press speed for each tablet press by subtracting the mean press speed from actual press speed and dividing by the standard deviation. Refer to supplementary information (Table S4) for the actual press speeds.

Formulation 1 ‘good flow’ and formulation 2 ‘fair flow’ displayed similar trends in tablet weight variability on the KG RoTab and Fette presses (Figure 2A,B and Figure 3A,B). Maximum tablet press speeds of 140,000 TPH could be achieved on the Fette press at high feeder speeds. The maximum tablet press speed predicted to achieve ≤1% RSD was 93,300 TPH (coded press speed 0.01) on the Modul P (Figure 3C).

**Figure 4:**
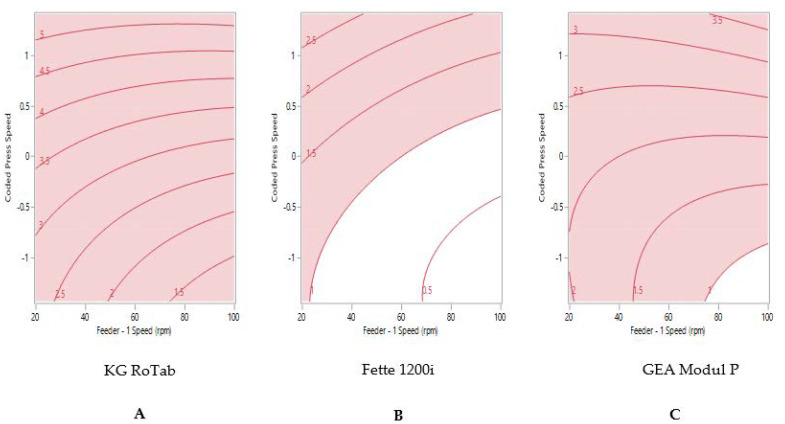
Two-dimensional contour profile showing optimum tablet press speed and feeder speed settings (uncoloured) for formulation 3 “passable flow” to achieve tablet weight variability ≤1% RSD on (**A**) KG RoTab, (**B**) Fette 1200i and (**C**) Modul P. Coded press speeds refer to standardized press speed for each tablet press by subtracting the mean press speed from the actual press speed and dividing by the standard deviation. Refer to supplementary information (Table S4) for the actual press speeds.

The sweet spot for formulation 3 ‘passable flow’ on each tablet press is shown in Figure 4. The process model did not predict that tablets weight variability ≤1% RSD could be produced on the KG RoTab (Figure 4A). Despite this formulation possessing the poorest flow characteristics amongst the three formulations, tablet weight variability ≤1% RSD was predicted for the Fette at press speed of less than 102,100 TPH with gradual increase in feeder speed from 23 rpm (Figure 4B). The sweet spot for the Modul P for formulation 3 showed that tablets with the target of ≤1% RSD were only achievable when the tablet press was run at lower press speed of 63,600 TPH (coded press speed of −0.88) and feeder speed of above 75 rpm (Figure 4C).

### 3.5. Model Validation

The process model was validated using retrospective compression data available for two formulations, i.e., a placebo formulation and an active tablet formulation. The active commercial formulation contained a combination of two different APIs. Firstly, the formulation flow behavior was benchmarked to the formulations used to develop the model. For this purpose, the permeability index was selected as the reference flow parameter, as it plays an important role in filling of materials in the die before compression as well as in compression packing efficiency [32]. The permeability of the formulations was measured as described in Section 2.3, and the results are shown in Table 5. Both formulations were classified as ‘fair flow’ due to the proximity of the measurements to the ‘fair-flow’ formulation used to establish the model (Table 5).

Retrospective data for these formulations produced on Fette 1200i and Modul P tablet presses at defined setting were compared to values predicted using the model for formulation 2 ‘fair flow’. The predicted and actual measured mean-weight RSD values are reported in Table 5. Overall, the model overestimated the tablet weight %RSD. The overestimation of the model may be due to differences in die filling behaviour between the placebo formulation, formulation 2 “fair flow”, validation formulations. However, an exception was observed for the placebo formulation at a tablet press speed of 90,000 TPH and a feeder speed of 30rpm (Table 5).

A possible explanation for the underestimation for this formulation at this setting may be a lack of sensitivity of the model to differences in blend flow behaviour at settings where blend flow into dies can be involved, e.g., combinations of relatively high press speed and low feeder speed. In this study, due to the unavailability of a comprehensive set of flow data for the retrospective blends used for validation, a single parameter, permeability, was used. Other parameters commonly employed to describe flow and electrostatics, suction fill behavior and environmental conditions (humidity), or combinations of parameters might better identify critical flow characteristics when benchmarking blends into categories for the model.

## 4. Discussion

The study presented demonstrates the development of a process model to predict tablet weight variability, expressed as %RSD, for direct compression formulations with varying flow properties, across pilot- and production-scale tablet presses. Similar to an earlier study by Razavi et al. (2020), the logarithm of %RSD was chosen as the response variable. The process model included linear, quadratic and interaction effects of input parameters on log(%RSD), $R_{adj}^{2}$ of 84.82% and $R_{pred}^{2}$ of 82.77%. The approach undertaken incorporated the tablet press model and formulation flow as categorical variables in the experimental design and feeder and press speed as numerical variables. The resulting model and formulation–tablet press-specific optimum process conditions provided a comprehensive overview of the behaviour of direct compression formulations with varying flow behaviour across a range of press types. The modelling approach used advances the conventional QbD approach, where process-related models and desired processing parameters are generally limited to a single tablet press model [33].

Earlier studies have focused on the effect of feeder design and tablet press control strategy on tablet weight variability and optimise designs to reduce tablet weight variability [20,33]. The objective of this study was not to compare the capability of different tablet press designs but to develop a modelling approach to predict how formulations would behave on tablet presses with standard feeder systems and control strategies employed routinely in an industrial setting. The modelling approach undertaken enables a company to predict tablet weight variability across tablet presses in their inventory for formulations with varying flow. While this study investigated three specific tablet press models, the approach taken is applicable to other tablet press designs or set-ups. For example, in this study the weight control strategy employed for Module P was mode 2 (pre-compression displacement at equal force). The model could be developed or expanded to include other weight control mechanisms on the Modul P such as mode 5 (main compression force measurement).

The model was developed with placebo formulations designed to exhibit different flow characteristics. Blend flow behaviour was classified categorically using established Carr’s index, Hausner ratio, angle of repose values [34]. The predictive capability of the model was validated using process and tablet weight data from active and placebo direct-compression formulations related to previous production-scale tablet press models. Blend permeability measurements were used to benchmark the validation blends to blend flow categories used in the model. Blend permeability was selected, as it was identified, together with compressibility, density, flowability, porosity and wall friction angle, as the most critical parameter related to die filling in a recent study by [11]. Overall validation showed the model has a low negative bias, shown in its predicted tablet weight variability for both tablet press models at the majority of settings investigated. This may be attributed to the shorter DoE run times that predicted higher weight variability, compared to data from validation formulation runs recorded after attaining steady state, which has a greater run time. However, for one formulation/press setting, the model showed a low positive bias. Understanding of the model’s prediction capability may be improved by using more blend characteristics to benchmark validation formulations to formulations employed in model development. The incorporation of additional blend characteristics to describe blend flow could elucidate further differences between blend die filling behaviours.

When applied in an industrial setting, the process modelling approach undertaken offers several benefits. During development, the model can be applied to predict possible production speeds following scale-up based on formulation flow behaviour measured during formulation development. Depending on the API loading requirements, it may be possible to reformulate the blend to improve formulation flow and thereby increase commercial production speeds. It is also interesting to note from the results of this study that it was not possible to achieve ≤1% RSD for formulation 3 ‘passable flow’ on the pilot-scale KG RoTab press which is used to develop formulations. However, when compacting this formulation on the production-scale Fette press, it was possible to produce tablets with ≤ % RSD at speeds as high as 100,000 TPH. The application of the model to understand differences in die filling behaviour, measured as tablet weight variability, between a development and a production-scale press can provide reassurance during process development that a formulation with ‘passable flow’ can be produced at production scale, despite not meeting the desirable specifications of tablet weight variability when produced at pilot scale. During commercial production, understanding differences in die filling behaviour between production-scale presses facilitates the selection of optimum feeder and press speed to suit formulation flow when transferring between presses. It can be also used to select a tablet press from an available inventory to maximise tablet production. Finally, the process modelling approach developed can streamline tech transfer activities between lab-scale and production-scale tablet presses. Utilising this approach has the potential to reduce process development time and thus bring new products to the market faster.

## 5. Conclusions

An empirical process model was developed to predict tablet weight variability, expressed as percentage relative standard deviation, for direct compression formulations with varying flow properties, across pilot- and production-scale tablet presses. The model was developed using a DoE approach, with press and feeder speed as numerical variables and press type and formulation type as categorical variables. Optimum processing conditions were established for each formulation, good, fair and passable flow, on each press to achieve tablet weight variability ≤1% RSD. The model developed was successfully validated using a placebo and an active formulation on two production presses. Blend permeability was used to benchmark validation formulations to model formulation categories. This allows the prediction of commercial-scale tablet weight control based on small-scale blend data during early-stage development. The modelling approach undertaken can be applied to an industrial setting to aid formulation development, scale-up and transfer of formulations between production presses.

## Figures and Tables

**Table 1:** Composition of placebo direct compression blends used to develop the process model and their flow properties.

	Placebo Blend Composition (% *w*/*w*)			
Components	Function	Formulation 1 Good Flow	Formulation 2 Fair flow	Formulation 3 Passable Flow
Microcrystalline Cellulose	Diluent	48.5	38.5	28.5
Lactose	Diluent	48.5	38.5	28.5
Sucrose Octaacetate	Anti-flow agent	0	20	40
Crospovidone	Disintegrant	1	1	1
Colloidal Silicon dioxide	Flow aid	1	1	1
Magnesium Stearate	Lubricant	1	1	1
Carr’s Compressibility Index		11.54	18.70	23.57
Hausner Ratio		1.13	1.23	1.31
Angle of repose (^0^)		27.16	37.75	43.56
Permeability at 15 kPa		109.32	54.66	21.33

**Table 2:** DoE feeder speed and tablet press speed levels and associated dwell times for each tablet press.

Tablet Press	Feeder Speed (rpm)	Tablet Press Speed (Tablets per Hour)	Dwell Time (Millisec)
KG RoTab	20	9600	66
	60	14,400	44
	100	19,200	33
Fette 1200i	20	28,000	33
	60	86,400	11
	100	140,000	7
GEA Modul P	20	46,000	35
	60	93,000	18
	100	140,000	12

**Table 3:** Summary of the process model statistical parameters.

Statistical Parameter	Value
*R* ^2^	0.8619
Adjusted *R*^2^	0.8482
Root Mean Square Error	0.2779
Predicted *R*^2^	0.8277

**Table 4:** Effect test summary of model terms (factors) and their interactions showing significant effects (*p* < 0.05) on the log-transformed tablet weight variability (%RSD).

Model Term	*p* Value
Tablet Press	<0.0001
Formulation Type	<0.0001
Feeder Speed	<0.0001
Press Speed	<0.0001
Tablet Press*Formulation Type	<0.0001
Press Speed*Press Speed	<0.0001
Formulation Type*Feeder Speed*Press Speed	<0.0001
Tablet Press*Feeder Speed*Press Speed	0.0035
Tablet Press*Formulation Type*Press Speed	0.0076
Formulation Type*Press Speed	0.0165
Feeder Speed*Feeder Speed	0.0481

* indicates interaction between the factors.

**Table 5:** Permeability values for validation formulations, validation process settings, process model, actual tablet weight variability (%RSD) for validation formulations and predicted tablet weight variability (%RSD) values calculated by the process model, benchmarking validation formulations to formulation-2 (fair flow).

Validation Formulation	Permeability at 15 kPa	Tablet Press	Tablet Press Speed (TPH)	Feeder Speed (rpm)	Actual Tablet Weight % RSD Validation Formulation	Predicted Tablet Weight % RSD Formulation-2	Bias (%RSD)
Placebo Formulation	54.21	Fette 1200i	60,000	20	0.74	0.84	−0.1
		Fette 1200i	90,000	30	1.50	1.09	0.4
		Fette 1200i	120,000	40	1.21	1.46	−0.25
Active Formulation	53.14	GEA Modul P	80,000	15	0.90	1.16	−0.26
		Fette 1200i	50,000	15	0.65	0.80	−0.15

## Data Availability

Data available on request due to restrictions.

## Supplementary Materials

The following are available online at https://www.mdpi.com/article/10.3390/pharmaceutics13071033/s1, Figure S1: Full response surface design DoE with 8 runs (green) and 4 centre points (blue). Figure S2: Effect summary of model terms (factors) and their interactions showing significant effects on the log transformed tablet weight variability (%RSD). Figure S3: Prediction profiler showing weight RSD as response (A). Medium flow formulation on Fette 1200i at coded press speed of −0.609 (60,000 TPH). (B) Passable flow formulation on Modul P at coded press speed of 0 (93,000T PH). Details shown in red font indicate input factors in the model and output responses. Profiles indicate the change in response as individual factors are altered. Table S1: Key technical specification of tablet press in this study with specific type of tooling. Table S2: Pre-compression force and main compression force applied to achieve the target tablet porosity of 15% for each formulation on each tablet press. Table S3: DoE run order of experiments for three different formulations on three tablet presses. The center points are shown in bold. Table S4: Actual tablet press speeds.
