# Supplementary material for: Process Model Approach to Predict Tablet Weight Variability for Direct Compression Formulations at Pilot and Production Scale
Source: Pharmaceutics. 2021 Jul 7;13(7):1033. doi: 10.3390/pharmaceutics13071033 (PMC8308976; doi:10.3390/pharmaceutics13071033)
Supplement: Supplementary file 1 [file pharmaceutics-13-01033-s001.zip › pharmaceutics-1244512-supplementary.pdf]

pharmaceutics

MDPI

Supplementary Materials: Process Model Approach to Predict Tablet Weight Variability for Direct Compression Formulations at Pilot and Production Scale

Raghu V. G. Peddapatla, Gerard Sheridan, Conor Slevin, Shrikant Swaminathan, Ivan Browning, Clare O'Reilly, Zelalem A. Worku, David Egan, Stephen Sheehan and Abina M. Crean

Table S1. Key technical specification of tablet press in this study with specific type of tooling.

Parameters	KG RoTab	Fette 1200i	GEA Modul P
**No of punch stations**	8	24	21
**Max tablet output per hour (TPH)**	19,200	140,000	140,000
**Fill weight control mechanism**	Main compression force measurement	Main compression force measurement	Pre-compression displacement at equal force
**Feed frame design (no. chambers)**	2 chambers	3 chambers	2 chambers
**Punch type**	D	B	D
**Punch Shape**	Shield (Triangular)		
**Tip edge width (mm)**	8.5		
**Perimeter of punch tip (mm)**	27.8		
**Cross sectional area of punch tip (mm^2)**	54.8		

Table S2. Pre-compression force and main compression force applied to achieve the target tablet porosity of 15% for each formulation on each tablet press.

Tablet Press	Formulation	Pre-Compression Force (kN)	Main Compression Force (kN)
KG RoTab	1	1	8.1
	2	1	4.8
	3	1	1.4
Fette 1200i	1	1	12.2
	2	1	10.5
	3	1	8.7
GEA Modul P	1	1	13.8
	2	1	10.5
	3	1	6.6

Table S3. DoE run order of experiments for three different formulations on three tablet presses. The center points are shown in bold.

Runs	Feeder Speed	Press Speed
1	**0**	**0**
2	-1	0
3	1	0
4	0	1
5	0	-1
6	**0**	**0**
7	-1	1
8	1	1
9	**0**	**0**
10	**0**	**0**
11	1	-1
12	-1	-1

Table S4. Actual tablet press speeds.

Coded Press speeds	Actual Tablet Press Speeds (TPH)		
	KG RoTab	Fette 1200i	GEA Modul P
-1	10990	44399	59611
-0.5	12695	64300	76306
0	14400	84200	93000
0.5	16105	104101	109695
1	17810	124001	126389

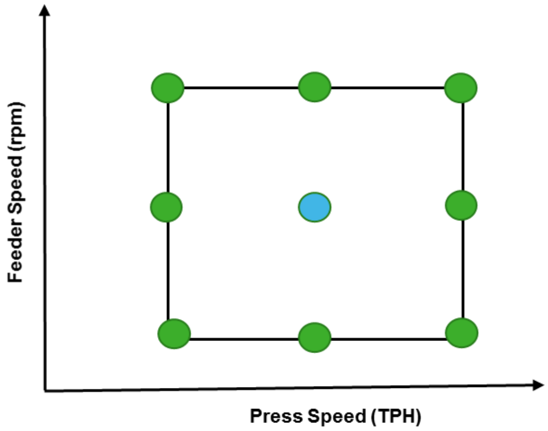

Figure S1. Full response surface design DoE with 8 runs (green) and 4 centre points (blue).

Effect Summary

Source	PValue
Formulation Type	0.00000
Tablet Press*Formulation Type	0.00000
Press Speed	0.00000
Feeder Speed (rpm)	0.00000
Tablet Press	0.00000
Press Speed*Press Speed	0.00000
Formulation Type*Speed (rpm)*Press Speed	0.00001
Tablet Press*Feeder - 1 Speed (rpm)*Press Speed	0.00346
Tablet Press*Formulation Type*Press Speed	0.00758
Formulation Type*Press Speed	0.01655
Feeder Speed (rpm)*Feeder Speed (rpm)	0.04806
Feeder Speed (rpm)*Press Speed	0.50428
Formulation Type*Feeder Speed (rpm)	0.51136
Tablet Press*Feeder Speed (rpm)	0.66025
Tablet Press*Press Speed	0.99360

Figure S2. Effect summary of model terms (factors) and their interactions showing significant effects on the log transformed tablet weight variability (%RSD).

Prediction Profiler

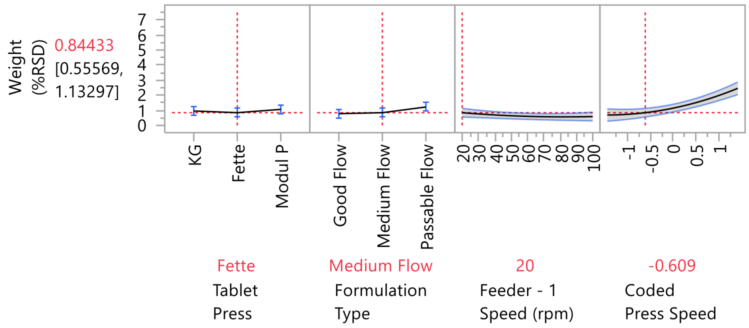

(A)

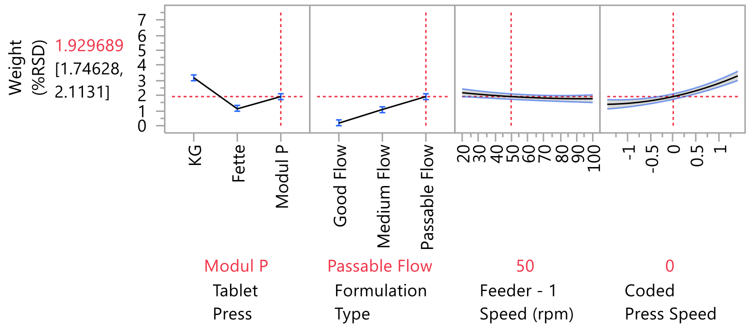

(B)

Figure S3. Prediction profiler showing weight RSD as response (A). Medium flow formulation on Fette 1200i at coded press speed of −0.609 (60000 TPH). (B) Passable flow formulation on Modul P at coded press speed of 0 (93000TPH). Details shown in red font indicate input factors in the model and output responses. Profiles indicate the change in response as individual factors are altered.
